# Fluctuations in Well-Being Based on Position in Elite Young Soccer Players during a Full Season

**DOI:** 10.3390/healthcare9050586

**Published:** 2021-05-14

**Authors:** Hadi Nobari, Maryam Fani, Elena Pardos-Mainer, Jorge Pérez-Gómez

**Affiliations:** 1 Department of Physical Education and Sports, University of Granada, 18010 Granada, Spain; 2 Department of Exercise Physiology, Faculty of Sport Sciences, University of Isfahan, Isfahan 81746-7344, Iran; 3 HEME Research Group, Faculty of Sport Sciences, University of Extremadura, 10003 Cáceres, Spain; Maryamfani.mf@gmail.com (M.F.); jorgepg100@gmail.com (J.P.-G.); 4 Sports Scientist, Sepahan Football Club, Isfahan 81887-78473, Iran; 5 Department of Biological Sciences in Sport and Health, Faculty of Sports Sciences and Health, Shahid Beheshti University, Tehran 1983963113, Iran; 6 Health Sciences Faculty, Universidad San Jorge, Autov A23 km 299, 50830 Zaragoza, Spain; epardos@usj.es

**Keywords:** non-functional overreaching, DOMS, fatigue, performance, monitoring

## Abstract

The current study surveyed weekly and daily variations of well-being ratings relative to the Hooper Index (HI): fatigue (wFatigue), stress (wStress), delayed onset muscle soreness (wDOMS), and sleep quality (wSleep) during a soccer season based on players’ positions. The full-season was divided into three meso-cycles: Early season, week (W)1 to W7; Mid-season, W8 to W13, and End-season, W14 to W20. Twenty-six young players participated in the study (age, 15.5 ± 0.2 years; height, 172.9 ± 4.2 cm; body mass, 61.4 ± 5.6 kg; body fat, 8.6 ± 2.9%; VO_2max_, 48.4 ± 2.4 mL.kg^−1^·min^−1^; maturity offset, 1.9 ± 0.3 years). Participants played in the same team and competed in Iran national under-16 competitions. Well-being status was monitored on training days using the HI questionnaire. The main result was a significant difference between well-being status 5 days before match day (MD) and 4 days before MD, compared to MD for all playing positions (*p* ≤ 0.001). The highest and lowest records occurred during End-season for wDOMS (strikers = 11.5 ± 8.4 arbitrary units (AU)), Early season (central defenders = 9.5 ± 0.7 AU) and for wFatigue (central midfielders = 11.4 ± 0.9 AU), and Early season (wide defenders = 9.7 ± 0.7 AU), respectively. Overall, the results showed a significant increase in wStress and wSleep for all players’ positions from Early- to End-season. The main application of this study is to make coaches aware of their players’ well-being fluctuations throughout the full season, especially in young elite soccer players, and to avoid injuries, overtraining, and overreaching as much as possible.

## 1. Introduction

In team sports, the continuous monitoring of training loads is required to ensure a proper recognition of the training stimulus on a team [1]. The purpose of training monitoring is to determine the biological and physical impact that training and games have on players [2]. On the other hand, playing at a high-level in soccer requires the use of intensive training to enhance and develop players’ fitness. During intense training periods, there is an increase in training loads (TL). As a result, athletes adapt rapidly to the requirements of the competitive period [3,4]. Besides, soccer has also been reported as an intense activity. High-intensity movements (e.g., sprints, accelerations, or decelerations) occur at crucial moments in soccer, such as ball contests, offensive or defensive acts, and goal-scoring occasions, and these intensive actions have an impact on a match’s outcome [5,6]. In addition, controlling the physiological and physical condition of players provides information about their individual needs. One of the main advantages of individual monitoring, based on position, is to recognize demands. Studies have shown that differences in anthropometric and fitness characteristics have made players more successful in different positions of the game, according to their body shape and size [7,8]. Roles and needs in each position represent different energy systems, as well as physiological and psychological demands. Soccer is known as a sport with random events and various movements, including walking, jogging, running, and sprinting in different directions, as well as specialized technical movements such as passing, shooting, and heading. Moreover, players in different positions have several functions required for that position [8]. As research has shown, defender players have more backward, lateral and explosive movements, such as jumping, compared to other positions, and these movements consume 20% to 40% more energy than running forward. Midfielders, on the other hand, run longer distances but with a lower or more moderate intensity than attackers and defenders. Attackers, like defenders, are more prone to explosive movements and sprints. Additionally, due to their role in scoring, they have more body-to-body contact and high physical strength [9]. The present evidence reflects the fact that specific physiological needs and anthropometric differences in several players have led to each young player being selected for their playing position, based on superior physical and better physiological function required for those positions [7]. Therefore, individual analysis and monitoring will determine the physiological needs of each player according to their position. In fact, to know the physical load and stress imposed on soccer players, according to their role and position during the match or training, is necessary for personal training protocols, and individual monitoring gives a wider view to coaches and players for better management in the prevention of injuries, and also to improve performance [9].

Several studies have shown that physiological changes during intensive soccer training are linked to psychological changes and disappointing performances [10,11,12,13]. Intense training may also have an influence on the physical factors that affect athletic performance, such as sleep and recovery quality, stress, fatigue, and muscle soreness [10]. The overall goal of training monitoring is to identify the biological and physical effects that training sessions and matches have on players [2]. Moreover, a precise TL monitoring may provide valuable information to improve the planning process and to reduce injury risk, so it will help coaches in the early detection of injuries, bad-overreaching, or overtraining [14]. Overreaching has been identified as a significant difficulty in sport, and continues to challenge both coaches and athletes in their attempts to achieve optimal sport performance. Indeed, non-functional overreaching (NFOR) occurs when athletes do not sufficiently respect the balance between training and recovery [15]. Specific daily reactions of the players, according to their role in play positions and physiological responses, can also change by consequences of TL [9]. As a result, it is important to be mindful of well-being indices such as sleep quality the night before, stress, fatigue, and delayed onset muscle soreness (DOMS). In fact, to determine the level of well-being of players, the Hooper Index (HI) has been introduced, which includes self-analysis questionnaires about the fatigue, stress, DOMS, and sleep quality of players [16,17].

In general, soccer players have different physiological needs depending on their performance and position in training or competition. Additionally, an individual analysis of different players is very important to achieve optimal performance and the best results [9]. Due to maturity conditions, physiological and physical changes in young players, for example, the intensity of the game decreases with age, and youths have a shorter competition duration. The distanced covered increases with age (top level senior: 11 km, U18: 9 km (17), and U12: 6.2 km). Senior soccer player also has a higher percentage of maximal heart rate (HR_max_) (professional senior: 93% of HR_max_ and U18: 82% of HR_max_). These age-related changes highlight the importance of monitoring TL in young athletes [7]. On the other hand, because young elite soccer player are at high level of physical preparation compared to their amateur peers, they may have a different perception of TL and well-being statuses [18,19]. Therefore, considering the physiological differences in young soccer playing positions, it is important to measure the well-being of all players during the week to monitor their internal TL. Besides that, most youth soccer teams used to train 3–5 times a week and play one match per week during the season [1]. Weekly load or micro-cycle monitoring demands help to ensure optimum performance in official competition and to avoid injuries [20]. Moreover, elite coaches try to prepare athletes with the suitable load that prevent injury, overtraining and NFOR through the meso-cycles in the season [21].

Additionally, based on previous studies that have shown physiological and physical differences among playing positions, our hypothesis is that the well-being conditions of different player’s positions during various meso-cycle varies, so their TL should be monitored individually; it can also be used to identify NFOR syndrome in young soccer player [8,15,22]. Although monitoring TL in different positions is important due to different physiological conditions, the wellness conditions in different soccer positions in young players have not been studied. Therefore, twofold objectives have been defined for this study: (1) To describe daily patterns and comparisons between weeks and MD in the well-being status, both during micro-cycles of competition for the overall team and by playing position; and (2) to analyze the weekly differences of fatigue, stress, sleep, DOMS, and well-being status between meso-cycles (Early, Mid- and End-season periods) by playing position for the overall team.

## 2. Materials and Methods

### 2.1. Participants

In this survey twenty-six elite young soccer players participated (mean ± standard deviation (SD); age, 15.5 ± 0.2 years; height, 172.9 ± 4.2 cm; weight, 61.4 ± 5.6 kg; body fat, 8.6 ± 2.9%; VO_2max_, 48.4 ± 2.4 mL·kg^−1^·min^−1^; peak height velocity (PHV), 13.6 ± 0.3 years; maturity offset, 1.9 ± 0.3 years). Players participated in Iran’s national under-16 competitions as part of the same team. To measure the difference between different positions, we distributed all players in the following manner: six wide defenders (WD) and wide midfielders (WM), five central defenders (CD) and central midfielders (CM), and four strikers (ST) [23,24]. The characteristics of these players are in Table 1. The inclusion criteria were: (i) players who participated in at least 90% of training seasons; (ii) players were not allowed to participate in another training plan along with this study; (iii) each player who did not participate in the match during the week practiced in a separate training session, without the ball or with small side games. The Ethics Committee of the University of Isfahan approved this article before reading it. Players and their parents signed the consent form, which was based on the Declaration of Helsinki, that is widely recognized as the cornerstone document on human rights.

### 2.2. Sample Size

F-test: a within-group factor in a repeated measure with five groups and three measurements was used to determine the sample size for this analysis, according to the statistical method employed. It revealed a 96.2 % success rate (actual power). With twenty players, the likelihood of rejecting the null hypothesis for well-being monitoring outcomes can be calculated.

### 2.3. Experimental Approach to the Problem

This study is a descriptive longitudinal for monitoring a soccer team’s full season. Players were monitored daily during the 20 weeks of the season. The whole season was divided into three meso-cycle according to the starting team’s competition schedule: Early season, W1 to W7; Mid-season, W8 to W13, and End-season, W14 to W20. To analyze the differences between the meso-cycles with and without playing position, all well-being variables were considered for analysis. The explanation of the typical micro-cycle pattern, and its corresponding analyses, were conducted considering only the data from those competition weeks with the most repeated training pattern; the data included only one match per week. Players had been using the Hooper questioners for the previous two years. Daily sleep, stress, fatigue, and DOMS data were collected to report changes in weekly wellness status (i.e., Hooper Questioner) [22,25]. To calculate the VO_2max_ of the players, an intermittent fitness test 30-15 (30-15_IFT_) was performed in the pre-season.

### 2.4. Anthropometric and Body Composition

To calculate standing and sitting heights, a Stadiometer was used, as well as “Seca model 813, UK” to measure the weight of each athlete. These data were used to identify the maturity offset and age at peak height velocity (PHV) of the subjects using the down formula [26] as follows: maturity offset = −9.236 + 0.0002708 (leg length × sitting height) − 0.001663 (age × leg length) + 0.007216 (age × sitting height) + 0.02292 (weight by height ratio), where R = 0.94, R2 = 0.891, and SEE = 0.592), and for leg length = standing height (cm) − sitting height (cm) was used. In addition, we calculated the body fat (BF)% of the seven-point method, and body density (BD) was determined using the Jackson and Pollock equation, while BD and BF% were assessed using Brozek’s formula [27]. All considerations, accuracies, and measurement methods were based on the same project and previous articles of the present study [20,23,28]. These measurements were taken in the morning by a person with more than 6 years of research experience in this scope of study [29,30,31].

### 2.5. Aerobic Power Test

To calculate the VO_2max_ and the readiness levels of the subjects, 30-15_IFT_ was performed. The following formula was used to determine VO_2max_ [32]: VO_2max_ (ml.kg^−1^·min^−1^) = 28.3 − (2.15 × 1) − (0.741 × 16 years) − (0.0357 × weight) + (0.0586 × 16 years × VIFT) + (1.03 × VIFT), where VIFT= is the final running speed. The test was performed according to the same project and previous articles of the present study [20,23,28].

### 2.6. How to Monitoring Well-Being Status

HI is a 7-point scale personality questionnaire that assesses well-being in relation to stress, fatigue, DOMS, and sleep quality/disorders [22,25,33]. The sum of the four subjective scores introduced the well-being status. Subjective scores on a scale of 1–7 were used to determine HI, ranging from “very close to zero” (point 1) to “very great” (point 7). Players were familiar with the scale for at least two years, having used the Hooper questionnaire. The questionnaire was administered 30 min prior to the training session or match. Before beginning any exercise, participants were asked to rate these variables on a Likert scale to determine general wellness indicators. To determine DOMS, players’ thigh muscles were contracted in their range of motion, and the amount of muscle pain was recorded [25,34]. Using a custom-designed program on a portable computer tablet, players were asked for their HI variables individually. By pressing the relative point on the mobile, the participants chose their HI rating for every object, which would then be transferred directly under the player’s account. This approach reduced the impact of factors such as peer pressure and copying other players’ HI ratings. Afterwards, this information was placed in an Excel file daily. The strength and conditioning coach of the team collected all these data. The following collected data were obtained: (i) weekly stress (wStress), (ii) weekly fatigue (wFatigue), (iii) weekly DOMS (wDOMS), (iv) weekly sleep (wSleep), and (v) weekly well-being status.

### 2.7. Statistical Analysis

Descriptive statistics are introduced as mean and SD. Shapiro–Wilk and Levene’s tests were executed for verifying data normality and homogeneity, respectively. Changes between the three meso-cycle were determined using a repeated-measures analysis of variance (ANOVA), followed by Bonferroni post hoc test for pairwise comparisons [(Group × periods) with compare (Group) and (Group × periods) with compare (periods)]. Partial eta squared (η_p_^2^) was calculated as effect size of the repeated-measures ANOVA. Similar procedures were used for analyzing the possible differences between every weekday and the MD in well-being status during a common competition micro-cycle [(Group × day) with compare (Group) and (Group × day) with compare (day)]. Hedge’s g effect size with 95% confidence interval was also calculated to characterize the magnitude of pairwise comparisons for between meso-cycles comparatives. The Hopkins’ thresholds for effect size statistics were used as follows: ≤0.2, trivial; >0.2, small; >0.6, moderate; >1.2, large; >2.0, very large; and >4.0, nearly perfect [35]. Significance level was set at *p* ≤ 0.05. The Statistical Package for Social Sciences (SPSS, version 25.0; IBM SPSS Inc, Chicago, IL, USA) was applied for computations. Calculated a priori assessment of power and sample size, and the statistical software (G-Power; University of Dusseldorf, Dusseldorf, Germany) was applied. The selected study design utilized F-test; ANOVA, Repeated Measures, Within Factors; Power α err probability of 0.05, power 1-β err probability of 0.95, and the number of groups and measurements were five and three, respectively.

## 3. Results

**Figure 1:**
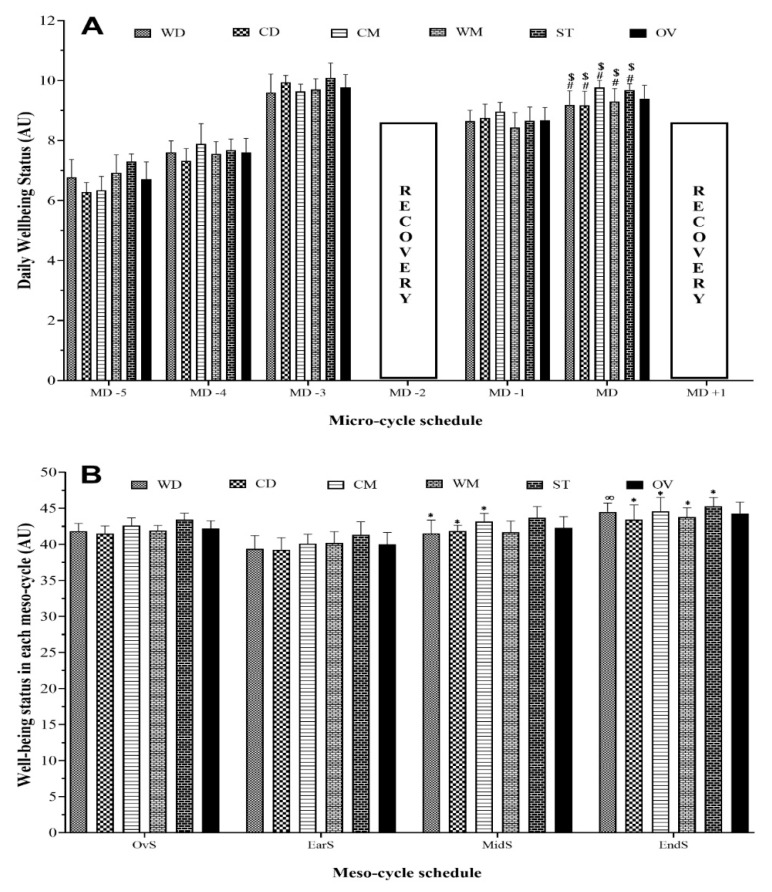
Micro-cycle (**A**) and meso-cycle (**B**) pattern and comparisons between each day (**A**) and each period (**B**) in well-being status (sum of Hooper questionnaire indexes) during a competition season by field position and overall team; WD, wide defenders; WM, wide midfielders; CD, central defenders; CM, central midfielders; ST, strikers; OV, overall team; MD, match day. In (**A**) ^#^ significant differences for *p* ≤ 0.05 compared to MD with MD_−5_; ^$^ Significant differences for *p* ≤ 0.05 compared to MD with MD_−4_. In (**B**) * represents a statistically significant difference compared to Early season (*p* ≤ 0.05); ∞ Represents a statistically significant difference compared to Mid-season (*p* ≤ 0.05).

Figure 1 displays the micro- and meso-cycles and comparisons between every weekday and the MD in the well-being variables during a main competition by playing position. The results of repeated-measures ANOVA were performed on two models. An analysis of differences between the well-being status between players’ positions was performed on a daily basis. There were no differences in the comparisons within weekdays of the overall team and playing positions of the players. A significant difference was found between well-being status in MD_−5_ (5 days before match day) and MD_−4_ (4 days before match day), compared to MD for all playing positions (*p* ≤ 0.001). MD_−4_ and MD^+1^ were not considered for analysis because well-being variables were not measured during recovery days. Thus, this point supposes a limitation of the study.

Figure 1B shows the highest and the lowest well-being status occurring in the End-season (ST = 45.3 ± 1.3 arbitrary units (AU)) and Early season (CD = 39.2 ± 1.7 AU), respectively. There was a significant increase in all playing position from Early to End-season. While there was no difference between Mid- to End-season, except for the WD playing position, which increased (*p* = 0.01).

**Figure 2:**
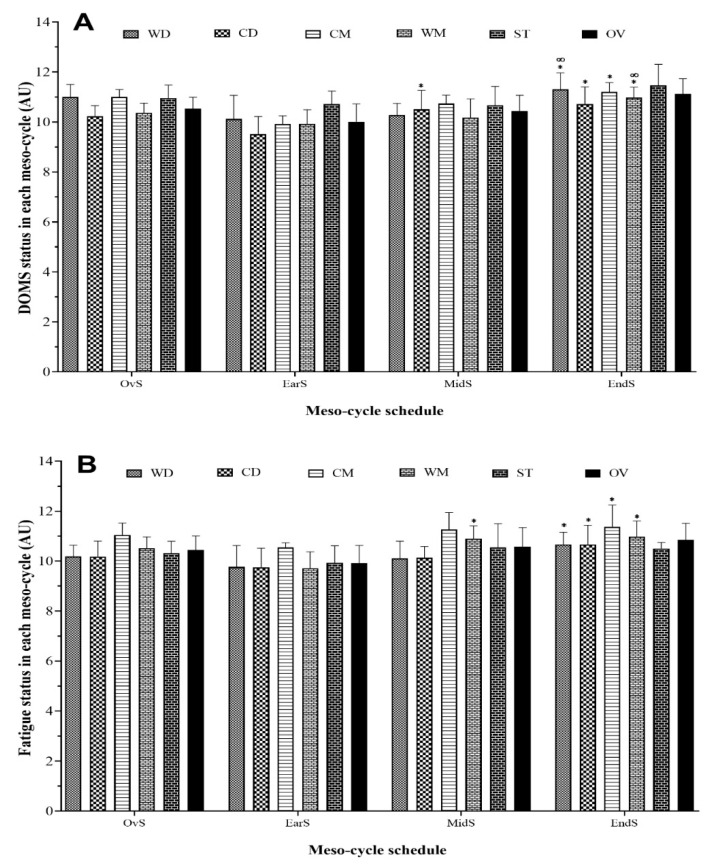
Meso-cycle of DOMS (**A**) and fatigue (**B**) pattern and comparisons between each period during a competition season by field position and overall team. WD, wide defenders; WM, wide midfielders; CD, central defenders; CM, central midfielders; ST, strikers; OV, overall team; DOMS, delayed onset muscle soreness. * Represents a statistically significant difference compared to Early season (*p* ≤ 0.05); ∞ Represents a statistically significant difference compared to Mid-season (*p* ≤ 0.05).

Figure 2 illustrates wDOMS and wFatigue variations between meso-cycles by playing position. The highest recorded occurred during the End-season for wDOMS (ST = 11.5 ± 8.4 AU) and wFatigue (CM = 11.4 ± 0.9 AU), In contrast, the lowest recorded occurred in the Early season for wDOMS (CD = 9.5 ± 0.7 AU) and wFatigue (WM = 9.7 ± 0.7 AU). There was a significant increase in all playing positions, except ST, from Early to End-season in wDOMS and wFatigue. There was no difference between the Mid- to End-season, except for the WM (*p* = 0.019) and WD (*p* = 0.003) playing positions, which increased in wDOMS.

**Figure 3:**
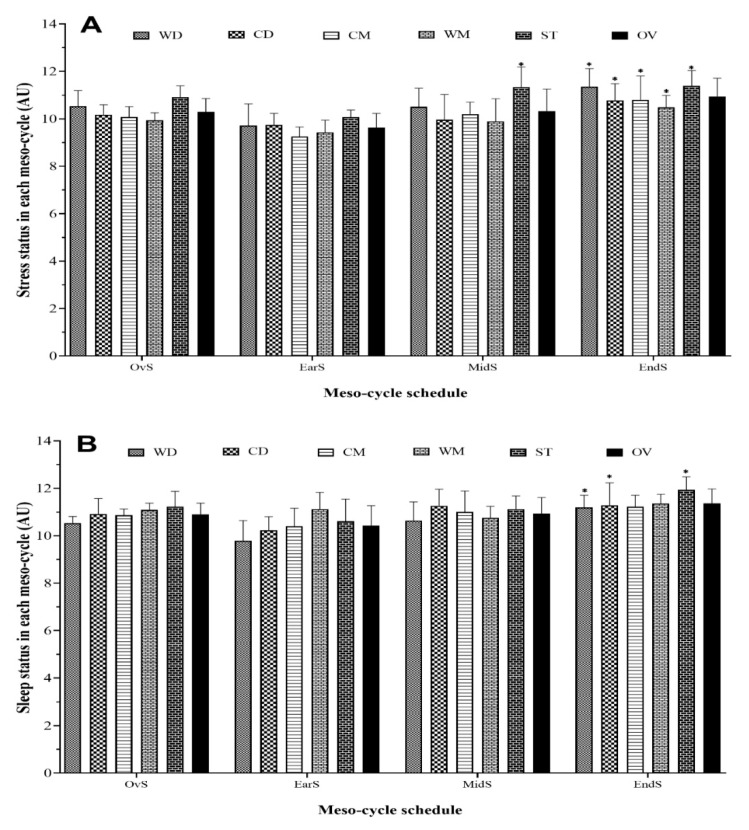
Meso-cycle of stress (**A**) and sleep (**B**) pattern and comparisons between each period during a competition season by field position and overall team. WD, wide defenders; WM, wide midfielders; CD, central defenders; CM, central midfielders; ST, strikers; OV, overall team; DOMS, delayed onset muscle soreness. * Represents a statistically significant difference compared to Early season (*p* ≤ 0.05).

The comparisons between different playing positions are displayed in Figure 3 for wSleep and wStress in each meso-cycle of the competition season. The highest recorded occurred during the End-season for wSleep (ST = 11.9 ± 0.6 AU) and wStress (ST = 11.4 ± 0.6 AU). In contrast, the lowest recorded occurred in the Early season for wSleep (WD = 9.8 ± 0.9 AU) and wStress (CM = 9.3 ± 0.4 AU). Overall, results showed there was a significant increase for all playing positions from Early to End-season in wSleep and wStress, except for CM and WM in wSleep. No difference was observed in the analysis between playing positions in the all well-being status variables within each meso-cycle.

Results of a repeated-measures ANOVA revealed differences between competition season meso-cycles in wSleep (*p* < 0.001, η_p_^2^ = 0.39), wDOMS (*p* < 0.001, η_p_^2^ = 0.53), wFatigue (*p* < 0.001, η_p_^2^ = 0.47), wStress (*p* < 0.001, η_p_^2^ = 0.54), and weekly well-being status (*p* < 0.001, η_p_^2^ = 0.71). Table 2 presents the pairwise comparisons between all in-season periods for wSleep, wDOMS, wFatigue, wStress, and weekly well-being status. Overall, a significant increase in all variables was observed in season meso-cycles, except for the wFatigue, compared to End- to Mid-season, and wSleep compared to Mid- to Early season (*p* > 0.05).

## 4. Discussion

The purpose of the present investigation was to identify well-being changes in micro- and meso-cycles based on positions in elite young soccer players. Twenty-six elite players participated in this study. The results of examining changes in players well-being showed that the highest and the lowest well-being status occurred during the End-season and Early season, respectively. There was a significant increase in all playing positions from Early to End-season. Indeed, the fluctuations of well-being in players of different positions due to physical and physiological needs were significant.

Sleep, stress, fatigue, and DOMS may be used to monitor training stimulus prescription, to determine well-being, to avoid NFOR, to avoid negative effects regarding training, and to avoid degradation in psycho-physiological functioning [36]. Numerous studies have shown that HI is one of the easiest and most accessible ways to monitor training intensity and load [22]. In the present study, we observed that the highest recorded occurred in the End-season for wDOMS and wFatigue. In contrast, the lowest recorded occurred in the Early season for both of them. We realized similar results for wSleep and wStress. Therefore, changes were noticeable at the Early season compared to the End-season, and the conditions of both were better at the Early season than at the End-season. Comparisons between all in-season periods for wSleep, wDOMS, wFatigue, wStress, and well-being status showed that, overall, a significant increase in all variables was observed in season meso-cycles, especially the in End-season in contrast with the Early season. As mentioned above, micro-cycle monitoring in the weekly training through the HI can provide useful information to coaches and players to avoid, as much as possible, the risk of injury, overtraining, and NFOR and, by applying the appropriate load in training, they will be able to achieve their best performances [37].

In addition, this is the first study that examines the changes in well-being in soccer players from different positions. In fact, physiological and functional differences in players with different positions cause differences in the conditions of well-being. For example, earlier research has shown that time-motion analyses of observed soccer matches demonstrated that CM and WM players covered a significantly greater distance than defenders and forwards [7,8,9]. As a result, we found in our research that the most well-being changes among the meso-cycles of the season are related to midfielders rather than strikers, which suggests that coaches need to pay attention to differences in players’ positions in recovery and training planning. Especially at the End-season compared to the Mid-season, the score of DOMS and fatigue was higher in WMs and CMs. In fact, these differences were observed in the high intensities of the match; at low intensities, there was no difference between different positions, and all players were at the same level [9]. In the present study, we also realized that most of the differences in different positions are related to the End-season, where the contending teams climbed and tried to obtain results, leading to intense activity, and Hooper scores were higher in various positions. Moreover, there is evidence that players in various positions have varying levels of psychological capacity. Attacking positions among soccer players have been found to have substantially greater emotional instability than most other players [38]. Strikers’ main target is to play as close to the opposing team’s goal as possible, making them the most likely to score goals. They actually control the ball, confuse the opponent, sprint, accelerate, and score at the ideal moment. Strikers have significantly higher confidence and motivation levels than defenders and midfielders, according to other reports. Even so, scoring goals seems to be the most essential matter of these players, which could also explain their sense of commitment among subjects in this situation [8,39]. As a result, it is hypothesized in this research that strikers’ obligations for scoring, as well as their positive attitude and high motivation to achieve their goals, have caused an increase in their stress levels. The main defensive action, on the other hand, is to defend the goal. Defenders must take the ball and prevent the opponent from advancing [40]. In general, the tasks of scoring and protecting the goal lead to determining the outcome of a match [9]. Additionally, perhaps these conditions have led to increased stress and sleep disorders for ST and defenders, especially in the most important matches at the end of the season. In the current study, the lowest scores were related to stress and sleep in the End-season compared to the Early season, and they were related to CM and WM.

This study also has limitations that need to be considered. First, present study was conducted only on a youth soccer team. It is better to consider and to compare different age groups. Secondly, we did not measure a recovery day; therefore, it is better to collect this information on the day of recovery because it will provide more accurate information about the wellness of the players during the week. For the final limitation of the current research, we can mention external TL monitoring, which was not included in the present study. Therefore, we suggest that by considering the external load indices such as covered distanced, acceleration, speed, etc. [41,42,43], with the well-being indices, a study with more accurate and more results can be conducted, in line with the present study. That can also affect the well-being status of players based on their playing positions. However, this is the first study to look at changes in the well-being of different players in micro- and meso-cycles during the season. Therefore, more studies should be performed on different sport teams and in different countries to generalize the results.

## 5. Conclusions

In the present study, according to the mentioned goals, we collected information about the well-being conditions of young soccer players during the soccer season. This information was measured by comparing the phases and different weeks of the season as micro- and meso-cycles. The well-being conditions of different players in different positions were also compared. Most of the well-being changes were related to the comparison of the Early season compared to the End-season, and the changes were more dramatic in the midfielders. Additionally, we observed a significant difference between the well-being status in MD_−5_ and MD_−4_, compared to MD for all playing positions; this is because of the recovery day. The overall purpose of investigating and examining these hypotheses is to make coaches more aware of the well-being of their players, and to consider the appropriate load during the days, weeks and months of the season to avoid, as much as possible, injuries, overtraining, and overreaching.

## Figures and Tables

**Table 1:** Absolute size anthropometric, body composition, and maturation of soccer player by playing positions.

Characteristics	Field Position									
	WD = 6N		CD = 5N		CM = 5N		WM = 6N		ST = 4N	
	Mean	SD	Mean	SD	Mean	SD	Mean	SD	Mean	SD
*Anthropometric*										
Age (years)	15.4	0.3	15.5	0.3	15.4	0.2	15.4	0.3	15.6	0.1
Height (cm)	175.8	4.0	174.4	5.0	169.8	1.3	171.2	2.9	172.8	5.3
Body mass (kg)	66.9	5.7	62.6	4.9	58.1	1.4	57.5	3.9	61.4	6.1
BMI (kg·m^2^)	22.2	1.5	21.0	1.2	20.1	0.4	19.3	1.2	20.8	1.9
VO_2max_ (mL·kg^−1^·min^−1^)	48.0	3.2	48.1	1.3	47.6	1.8	49.4	2.7	49.1	2.9
Career (years)	6.0	1.5	7.4	1.1	6.0	1.4	5.3	1.4	6.5	1.9
*Maturations* (years)									
PHV	2.1	0.2	1.8	0.4	1.8	0.1	1.7	0.4	1.9	0.2
Maturity offset	13.4	0.4	13.6	0.4	13.6	0.2	13.7	0.4	13.6	0.3
*Body compositions*										
BF%	8.2	2.2	10.3	3.8	10.4	3.3	6.1	1.9	8.6	0.6
BF (kg)	5.6	1.8	6.4	2.4	6.0	1.9	3.5	1.1	5.3	0.8
LBM (kg)	61.4	4.1	56.2	5.6	52.1	2.3	53.9	3.9	56.1	5.3

SD, standard deviation; WD, wide defenders; WM, wide midfielders; CD, central defenders; CM, central midfielders; ST, strikers; BMI, body mass index; VO_2max_, Maximal oxygen consumption; PHV, peak height velocity; BF, body fat; LBM, lean body mass.

**Table 2:** Comparative between competition season meso-cycles, considering well-being variables.

Variables	Season Period		Comparative	Mean Difference (95% CI)	*p*	Hedge’s *g* (95% CI)
**wDOMS (AU)**	Ear-S	10.0 (0.7)	Ear-S vs. Mid-S	0.4 [0.1 to 0.8]	**0.05**	0.6 [0.04 to 1.1]
	Mid-S	10.4 (0.6)	Ear-S vs. End-S	1.1 [0.7 to 1.5]	**<0.001**	1.5 [0.9 to 2.2]
	End-S	11.1 (0.6)	Mid-S vs. End-S	0.7 [0.3 to 1.0]	**<0.001**	1.0 [0.4 to 1.6]
**wFatigue** (AU)	Ear-S	9.9 (0.7)	Ear-S vs. Mid-S	0.7 [0.3 to 1.1]	**0.003**	0.8 [0.3 to 1.4]
	Mid-S	10.6 (0.8)	Ear-S vs. End-S	0.9 [0.5 to 1.3]	**<0.001**	1.2 [0.7 to 1.8]
	End-S	10.8 (0.7)	Mid-S vs. End-S	0.3 [0.1 to 0.7]	0.283	0.3 [0.2 to 0.9]
**wStress** (AU)	Ear-S	9.6 (0.6)	Ear-S vs. Mid-S	0.7 [0.3 to 1.1]	**0.003**	0.8 [0.3 to 1.4]
	Mid-S	10.3 (0.9)	Ear-S vs. End-S	1.3 [0.9 to 1.7]	**<0.001**	1.8 [1.1 to 2.4]
	End-S	10.9 (0.8)	Mid-S vs. End-S	0.6 [0.1 to 1.1]	**0.036**	0.7 [0.1 to 1.2]
**wSleep** (AU)	Ear-S	10.4 (0.9)	Ear-S vs. Mid-S	0.5 [0.1 to 0.9]	0.098	0.6 [0.05 to 1.2]
	Mid-S	10.9 (0.7)	Ear-S vs. End-S	0.9 [0.5 to 1.4]	**<0.001**	1.2 [0.6 to 1.8]
	End-S	11.4 (0.6)	Mid-S vs. End-S	0.4 [0.1 to 0.8]	**0.034**	0.6 [0.1 to 1.2]
**wWS** (AU)	Ear-S	40.0 (1.7)	Ear-S vs. Mid-S	2.3 [1.4 to 3.2]	**<0.001**	1.3 [0.7 to 1.9]
	Mid-S	42.3 (1.6)	Ear-S vs. End-S	4.3 [3.4 to 5.2]	**<0.001**	2.5 [1.7 to 3.2]
	End-S	44.3 (1.6)	Mid-S vs. End-S	2.0 [1.1 to 2.9]	**0.001**	1.2 [0.6 to 1.8]

AU, arbitrary units; CI, confidence interval; wSleep, weekly sleep in AU; wDOMS, weekly muscle soreness in AU; wFatigue, weekly fatigue in AU; wStress, weekly stress in AU; wWS, weekly well-being status (sum of Hooper questionnaire indexes) in AU; Ear-S, Early season period; Mid-S, Mid-season period; End-S, End-season period; *p*, *p*-value at alpha level 0.05; Hedge’s g (95% CI), Hedge’s g effect size magnitude with 95% confidence interval. Significant differences (*p* ≤ 0.05) are highlighted in bold.

## Data Availability

The datasets used and/or analyzed during the current study are available from the corresponding author on reasonable request.
